# Stepwise external wrapping procedure for Stanford type A aortic dissection in extremely high-risk patients: case reports

**DOI:** 10.1186/s13019-020-01142-x

**Published:** 2020-06-12

**Authors:** Yoshihiro Suematsu, Satoshi Nishi, Daisuke Arima, Akihiro Yoshimoto

**Affiliations:** Department of Cardiovascular Surgery, Tsukuba Memorial Hospital, 1187-299 Kaname, Tsukuba, Ibaraki 300-2622 Japan

**Keywords:** Type a acute aortic dissection, Stepwise external wrapping, High-risk patients

## Abstract

**Background:**

Acute aortic dissection (AAD) is a rare, but a life-threatening condition which can lead to coronary, brachiocephalic or branch vessel malperfusion, as well as aortic valve insufficiency, or aortic rupture. Mortality of surgical treatment in high-risk or elderly patients with Type A Acute aortic dissection (TAAAD) still remains high, and treatment for such patients remains controversial.

**Case presentation:**

A new surgical approach which entails “stepwise external wrapping (SEW)” using a zero-porosity artificial graft was developed in extremely high-risk patients with TAAAD. Herein, we described its surgical details and showed two representative cases which was successfully done.

**Conclusions:**

Our SEW procedure is a feasible alternative to conventional graft replacement for TAAAD in extremely high-risk or aged patients, although the gold standard consists of surgical replacement of the dissected aorta. (129 words).

## Background

Acute aortic dissection (AAD) is a rare, but a life-threatening condition which can lead to coronary, brachiocephalic or branch vessel malperfusion, as well as aortic valve insufficiency, or aortic rupture [1]. The International Registry of Acute Aortic Dissection (IRAD) reported the overall surgical mortality for type A AAD (TAAAD) improved from 25 to 18% form advances made in surgical techniques, anesthesia, and perioperative medical management. However, mortality of surgical treatment in high-risk or elderly patients with TAAAD still remains high and unchanged over the time, and treatment for such patients remains controversial [2].

We developed a new surgical approach which entails “stepwise external wrapping (SEW)” using a zero-porosity artificial graft. Herein, we reported two cases with major preoperative comorbidities and described surgical details.

## Case presentation

From June 2016 to March 2017, 2 patients with TAAAD underwent SEW procedure of the ascending aorta. Institutional review board approval was provided before publication of this article and reporting of the information. Informed consent was obtained by all patients or patients’ family in cases in which the patient was unconscious. Patient 1 was an 83-year-old man with tamponade and cardiogenic shock. Patient 2 was a 78-year-old woman also suffering from shock vitals and preoperative coma. A computed tomographic scan showed that entire aorta was dissected. Echocardiography confirmed pericardial effusion and no severe aortic regurgitation in both patients.

**Fig. 1:**
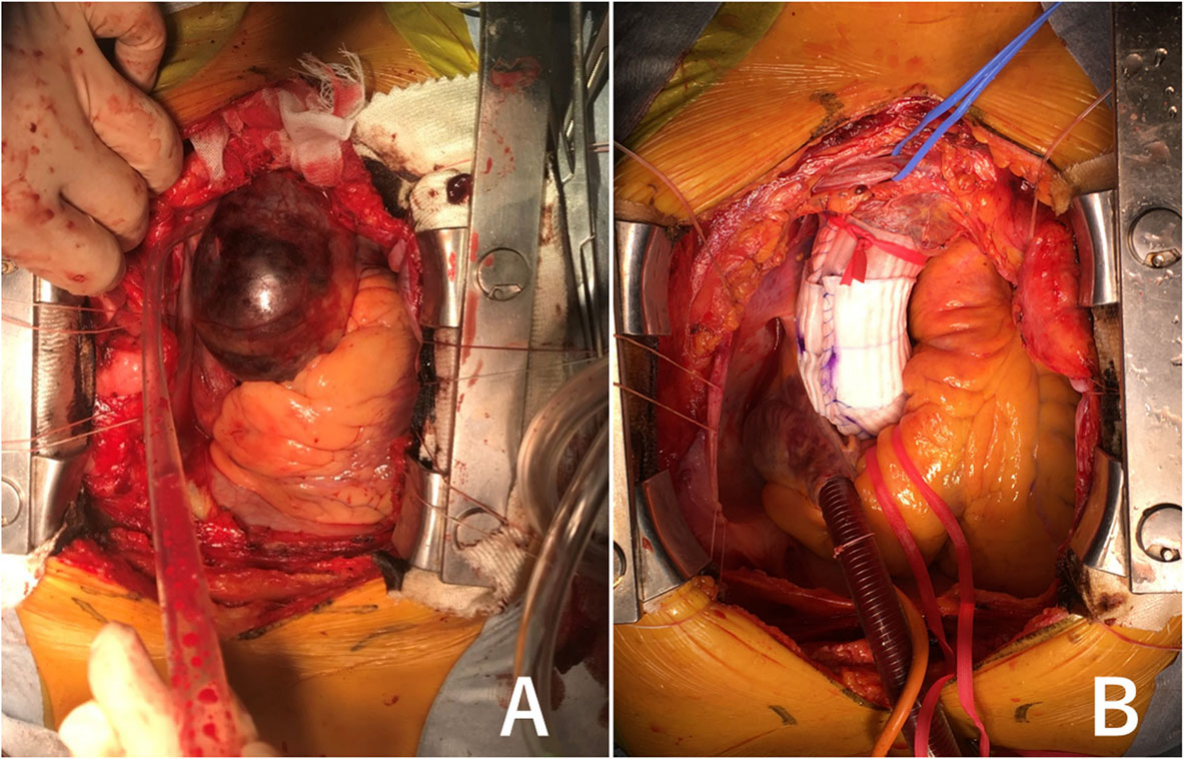
Operative Schema of Stepwise External Wrapping in Stanford type A acute aortic dissection

**Fig. 2:**
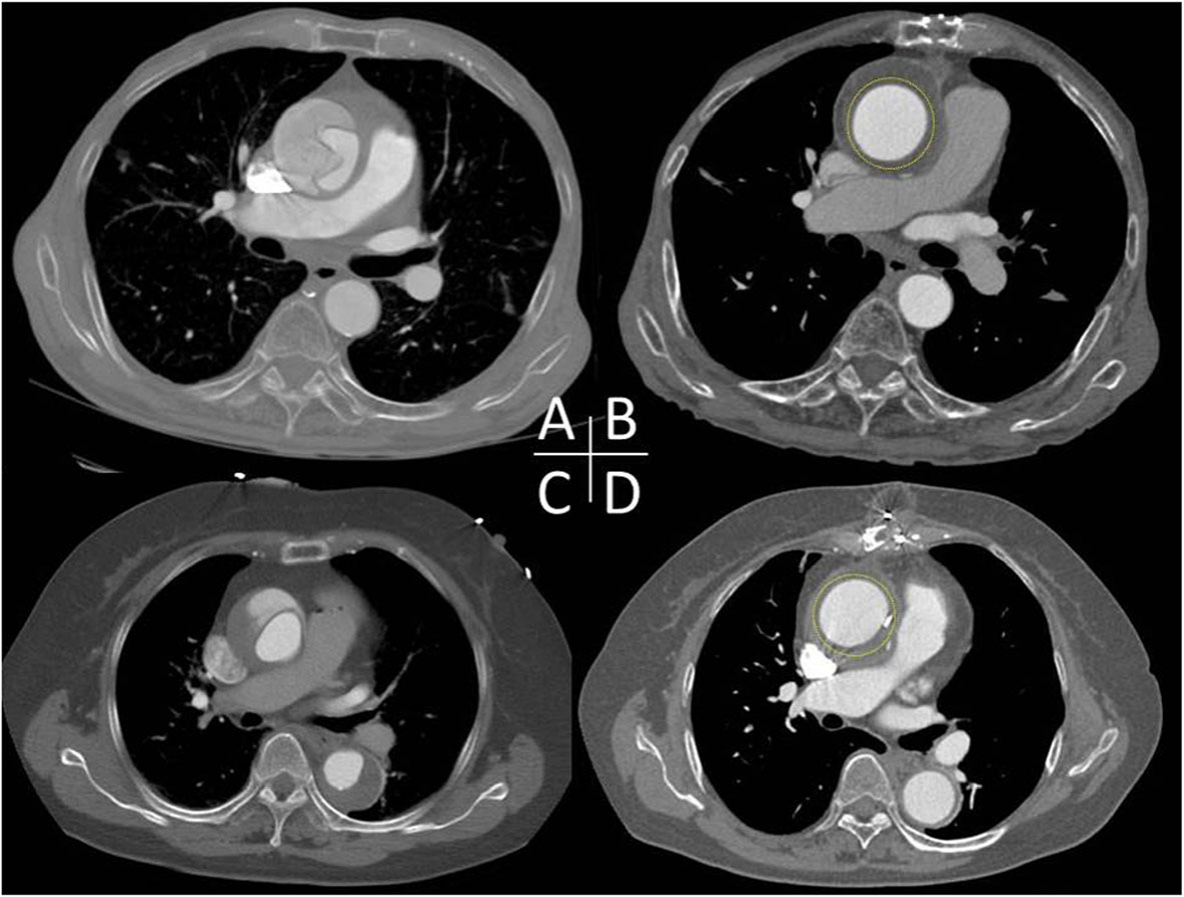
**a** and **c** Preoperative computed tomographic scan. **b** and **d** Follow-up postoperative computed tomographic scan after stepwise external wrapping procedure, demonstrating disappearance of false lumen and complete remodeling of the ascending aorta. Yellow dot showing line of the artificial graft

Median sternotomy was made and cardiopulmonary bypass was implemented through femoral arterial cannulation and single two-stage venous cannulation via right atrial appendage. The ascending aorta was carefully separated from the pulmonary artery trunk and the right pulmonary artery. Most care was taken during the dissection to avoid tearing the false lumen of the dissected aorta. Pieces of artificial graft was tailored, placed around the aorta from the coronary ostia up to the innominate artery in a stepwise fashion, and approximated with a running suture of 4–0 Prolene (Ethicon, Somerville, NJ) to tightly wrap the dissected ascending aorta (Fig. 1). We used Triplex graft® (Vascutek Terumo, Tokyo, Japan) as an artificial graft, which has the remarkable characteristics of excellent impermeability without biologic material coating [3]. The reason why we use separate piece of graft is for the snug fitting against the curved nature of the ascending aorta and size discrepancy between the proximal and distal ascending aorta, which can lead to prevent graft migration. We calculated the size of the graft so that the diameter of the wrapped aorta would measure around 40 mm to the dimensions of the patient and the preoperative CT scan. The aim was to significantly reduce the diameter of the aorta and maximize opposition between the false and true lumen. The overall operation times and cardiopulmonary bypass times were 91 and 31 min (Patient 1), and 80 and 23 min (Patient 2), respectively. There was no operative death. The postoperative hospital stays (10 and 8 days) were uneventful. At follow-up (38 and 30 months), the patients were doing well and were self-catering. Follow-up operative computed tomographic scans demonstrated a single lumen with the reapplication of the false lumen in the wrapped ascending aorta, and the absence of dissection or aneurysmal change in the entire aorta, in both patients (Fig. 2).

## Discussion

The concept of external wrapping surgery is not new, and it was first reported by Poppe JK for successful treatment of syphilitic aneurysms of the thoracic aorta in 1946 [4]. Subsequently, along with technological improvement of vascular prosthesis and advancement of peri- and post-operative management, graft replacement for acute aortic dissection became the gold standard therapy and thus wrapping surgery was abandoned. This strategy with currently conventional graft replacement carries an acceptable early mortality rate and provides good long-term outcome among survivors. However, despite technological improvements, the in-hospital mortality of patients that are high-risk, or aged, or unfit for surgical repair remained unchanged at approximately 60% [2].

Recently, Demondion P et al. reported a less invasive approach consisting of off-pump wrapping of the dissected ascending aorta with favorable short and midterm results [5]. There were several different points between their report and ours. One is use of cardiopulmonary bypass (CPB). Their paper emphasized an off-pump approach which contributed to a less invasive surgery. However, dissected aorta is extremely fragile, and there was a high possibility of aortic rupture or injury of the pulmonary artery during separation of the aorta from the pulmonary artery. Second, they are using Teflon plaque for wrapping, but it was very thick and with low competence of handling. Also, there is the differences in size between the proximal and distal ascending aorta, and curved configuration of the ascending aorta. To fit this configuration of the aorta, a single sheet is inappropriate.

In the present cases, there was no incidence of brain injury, re-exploration for bleeding, renal failure, respiratory failure, or hospital death among the sick patients who underwent SEW. There are many advantages in SEW procedure over conventional graft replacement. SEW procedure enables us to significantly shorten the CPB and operative durations. In high risk patients, it is believed shorter operation times provide better outcomes by way of reducing the surgical stress. Since SEW procedure does not require deep hypothermia the inherent risks and harm associated with CPB on coagulation system, lungs, and other organs is avoided. In addition, no brain protection or cardiac arrest was necessary. These advantages finally helped to prevent severe complications in treating high-risk patients conventionally.

## Conclusions

In conclusion, our SEW procedure is a feasible alternative to conventional graft replacement for TAAAD in extremely high-risk or aged patients, although the gold standard consists of surgical replacement of the dissected aorta. Further careful follow-up care will be needed.

## Data Availability

Yes

## Abbreviations

AAD: Acute aortic dissection
TAAAD: Type A Acute aortic dissection
IRAD: The International Registry of Acute Aortic Dissection
SEW: Stepwise external wrapping
CPB: Cardiopulmonary bypass
